# Maternal protein restriction affects the differentiation of cells in the epididymal epithelium lining of 44-day-old rats

**DOI:** 10.1242/bio.060080

**Published:** 2025-06-06

**Authors:** Fábio Colonheze, Marilia Martins Cavariani, Bruno Cesar Schimming, Talita de Mello Santos, Luiz Gustavo de Almeida Chuffa, Raquel Fantin Domeniconi

**Affiliations:** ^1^ Graduate Program in Health and Aging, Marilia Medical School, Marilia, SP 17213-000, Brazil; ^2^ Department of Structural and Functional Biology, Institute of Biosciences of Botucatu, São Paulo State University (UNESP), São Paulo 18618-689, Brazil

**Keywords:** AQP9, ATPase, KI-67, TP63, Epididymis, Protein restriction

## Abstract

Maternal protein restriction delays the differentiation of epididymal mesenchymal cells in newborn rats. However, it is unclear whether this delay persists until the full differentiation of the epididymal epithelium at 44 days postnatal. Thus, this study aimed to assess the impact of maternal protein reduction on 44-day-old rats’ epididymal epithelium differentiation, following up on the observed delay in newborn animals. Pregnant rats were randomly divided into groups receiving normal-protein (NP: 17% protein) or low-protein (LP: 6% protein) diets during gestation and lactation. On postnatal day (PDN) 44, male offspring were euthanized, and the epididymis (NP *n*=10, LP *n*=10) was processed according to immunohistochemical techniques for the detection of aquaporin 9 (AQP9), KI-67, TP63, and ATPase. LP rats showed a decrease in the intensity of the AQP9 reaction, an increase in cellular proliferation in the initial segment and corpus of the epididymis, an increase in basal cells in the caput and corpus epididymis, and an increase in ATPase-positive clear cells in the cauda region. These findings demonstrate that maternal protein restriction impacts cell differentiation in the epididymal epithelium of 44-day-old rats, persisting even with a normal-protein diet after weaning.

## Teaser

**Summary:** Even after post-weaning nutritional recovery, maternal protein restriction impairs epididymal epithelial cell differentiation in 44-day-old rats - an age at which full differentiation is normally expected.

## INTRODUCTION

Maternal nutrition has a pivotal impact on development and fetal health, since the fetus depends on the mother to obtain nutrients during gestation and lactation (McArdle et al., 2006; Jazwiec and Sloboda, 2019; Lindsay et al., 2019). According to McArdle et al. (2006), maternal nutrition is capable of influencing fetal health, and the consequences of inappropriate nutrition of the fetus may persist into adulthood. Studies reported that the component and the quality of the maternal diet during critical periods of embryonic or fetal development may remodel the offspring's genome in the uterus and epigenetic alterations induced in this period of life may permanently alter the phenotype of the adult organism, making it vulnerable to a range of diseases (Fleming et al., 2015; Jiang et al., 2014; Masuyama and Hiramatsu, 2012).

Chen et al. (2009) suggested that maternal protein restriction can affect major metabolic pathways implicated in regulation of lifespan at a young age, which may explain the impact of maternal diet on longevity. Moreover, maternal protein restriction can affect male offspring, changing their phenotype in a range of ways including reduction of the anogenital distance (Rinaldi et al., 2013; Cavariani et al., 2022), alterations in the serum concentration of testosterone, estradiol and dihydrotestosterone (DHT) (Zambrano et al., 2005; Teixeira et al., 2007; Rinaldi et al., 2013), delay in the development of the germinal epithelium and in the differentiation of Sertoli cells (Rodríguez-González et al., 2012), delay in the onset of puberty in adult animals (Rodríguez-Gonzáles et al., 2014; Toledo et al., 2011; Zambrano et al., 2005), and delay in the beginning of epididymis postnatal development (de Mello Santos et al., 2019).

Regarding the sperm alterations, it was observed that maternal protein restriction caused alterations mainly associated with the functions of the epididymis, such as sperm motility, viability and concentration, as well as increasing the number of spermatozoa with abnormalities, particularly in the flagellum and midpiece, and presence of cytoplasmic droplet (Rodríguez-González et al., 2014; Toledo et al., 2011). Cavariani et al. (2022) claimed that the insufficient supply of proteins in early life changes the structure and functioning of the epididymis in important periods of postnatal development. Even though studies have shown the effects of protein restriction related to epididymis functions, the causes of such alterations have not yet been clarified completely.

Recent results obtained by our research group show that maternal protein restriction changes the epididymal structure of the offspring rats, affecting specifically the dynamics of luminal fluids and the angiogenesis in important stages of the epididymis development (Cavariani et al., 2019). In this sense, maternal protein restriction impairs the structure and functioning of the developing epididymis, since the expression of proteins associated with regulation, development and maintenance of the epididymis is altered in an age-dependent manner (Cavariani et al., 2022). These findings could explain the alterations in the epididymis related to motility, viability, and concentration of the spermatozoa, which were described previously.

The epididymis is an androgen-dependent organ that is responsible for sperm maturation, transport, protection, concentration and storage. During the epididymal transit, the spermatozoa are subject to a continually changing luminal environment modified by the secretion and endocytic activities of the cells of the epididymal epithelium (Hermo and Robaire, 2002). The epididymis is lined by a pseudostratified epithelium and the epididymal cellular population is composed of principal, basal, apical, clear, narrow, and halo cells (Hermo and Robaire, 2002; Robaire and Hinton, 2015; Breton et al., 2016; Schimming et al., 2021). These specialized epididymal epithelial cells establish a unique luminal environment for the maturation and storage of spermatozoa (Breton et al., 2016).

The different epididymal cell types can be evidenced according to their morphological characteristics and also by the expression of proteins such as aquaporin 9 (AQP9), KI-67, TP63, and ATPase. AQP9 has been used as a marker for principal cells (Hermo et al., 2008). In addition to AQP9, other cell markers have been used to identify some epididymal cell types, such as KI-67, TP63 and ATPase, respectively marking proliferating cells, basal cells and clear cells (Kumar and Tanwar, 2017). Thus, it is possible to identify if the cell types are present and if they show a normal pattern of distribution along the different regions of the epididymal epithelium.

The evaluation of the TP63 protein can provide important information about the epididymal epithelium. This protein is used to label basal cells from different tissues of mice and humans. The TP63 is a homologous protein derived from TP53, that is, it has synergistic and/or antagonistic actions to the TP53 protein, whose function is tumor suppression. Blocking the cycle or apoptosis prevents the disordered proliferation of cells that have suffered some type of damage (El-Deiry, 1998; Yang et al., 1998). The KI-67 is a nuclear protein expressed in all phases of the cellular cycle. Studies suggest its importance in the synthesis of ribosomes during cellular division, in addition to the relationship with the fibrillar components and the nucleolus. In this way, it is used for labeling proliferating cells in their normal or tumor state (MacCallum and Hall, 2000; Rapidis et al., 2009).

ATPase is mainly expressed in the apical region of clear cells, which are responsible for modulating the pH of several biological systems. In the male genital system, these cells are responsible for the acidification of the lumen, which is important for the process of sperm maturation and storage. This function occurs through the cAMP-dependent signal transduction pathway, which allows cells to detect and modulate pH (Pastor-Soler et al., 2003).

Considering that the study of the effects of maternal protein restriction model on the differentiation of epididymis epithelial cells and its influence on the epididymis development appears to be relevant. This study aimed to evaluate the impact of maternal protein restriction on the structure and cellular differentiation of the epididymis epithelium in young rats investigating the expression of AQP9, KI-67, TP63 and ATPase, thus understanding whether the delay in the differentiation of mesenchymal cells observed in the beginning of the postnatal period, persisted around 44 days of postnatal life.

## RESULTS

Immunolocalization of AQP9, KI-67, TP63 and ATPase was observed in epithelial cells of the initial segment (IS), caput, corpus and cauda of the epididymis.

**Fig. 1.**
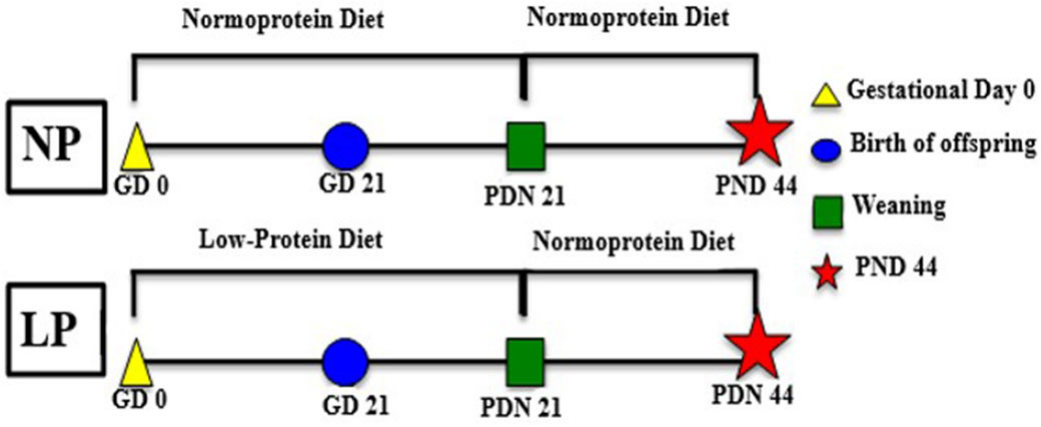
**Experimental design.** Pregnant rats received a normal-protein diet (NP group) or a low-protein diet (LP group) *ad libitum* from GD 0 until PND 21 (gestation and lactation periods). After weaning, male pups from both groups (NP and LP) received the NP diet for rodents until PND 44, when they were euthanized.

### AQP9 immunoreactivity in epididymal regions of NP and LP group animals

**Fig. 2.**
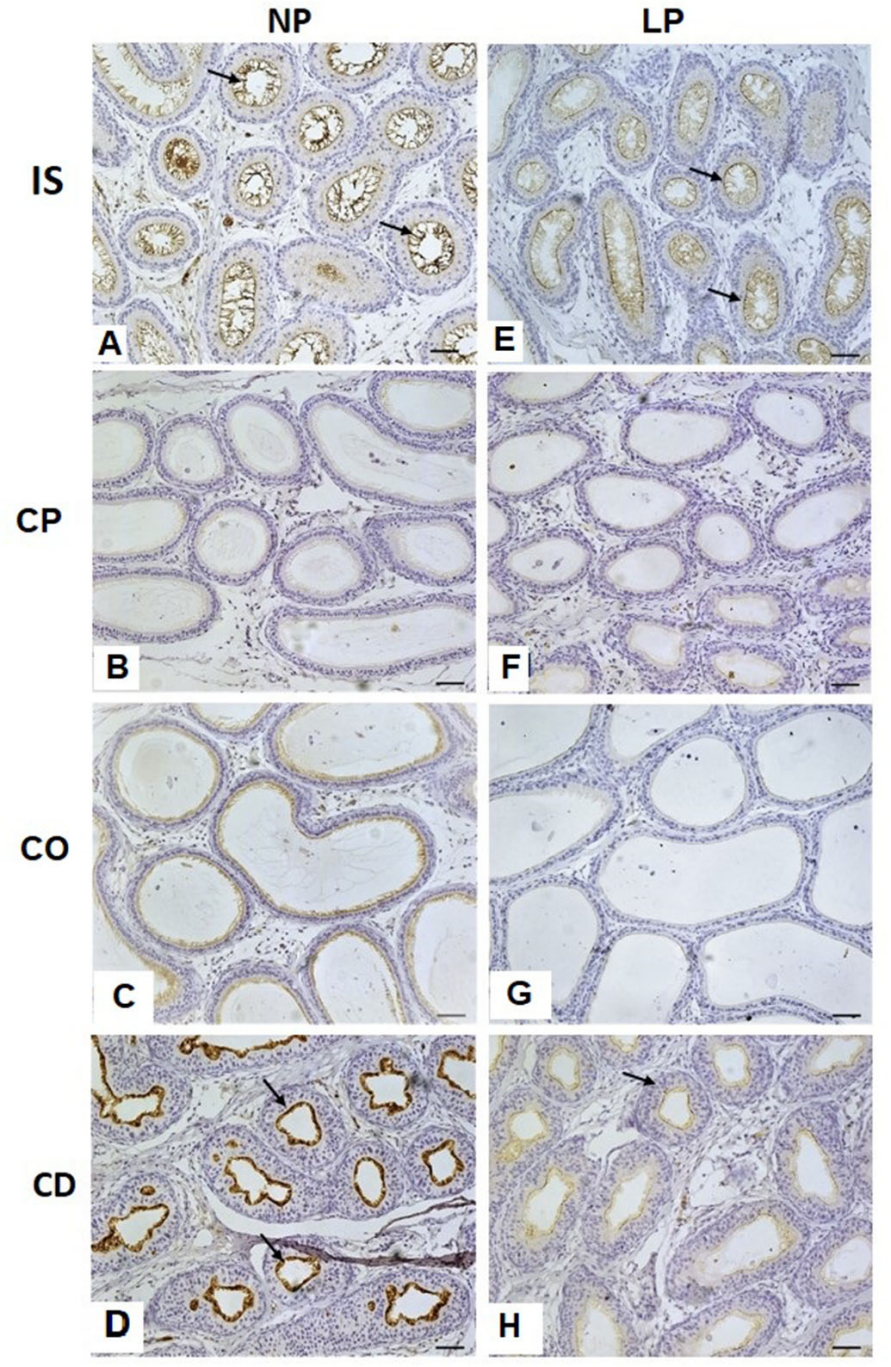
**Immunolocalization of AQP9 in the rat epididymis at 44 days old.** NP, *n*=5 and LP, *n*=5 groups. Arrows indicate the positive immunoreaction for AQP-9. Scale bar: 50 µm. IS, initial segment; CP, caput epididymis; CO, corpus epididymis; CD, cauda epididymis.

Strong AQP9 immunoreactivity was observed in the apical region of the principal cells in the IS of the epididymis in the NP group animals. There was a decrease in AQP9 immunodetection towards the caput region, which became intense once more in the corpus and cauda regions of the epididymis. In the comparative analysis between groups NP and LP it was possible to observe, through the immunohistochemistry reaction, that the intensity of AQP9 in the initial segment and in the cauda of the LP group was lower than in the NP-group animals. The other epididymal regions (caput and corpus) of the LP-group animals did not show AQP9-positive reactivity (Fig. 2).

### KI-67 marker staining for cell proliferation in epididymal epithelial cells

**Fig. 3.**
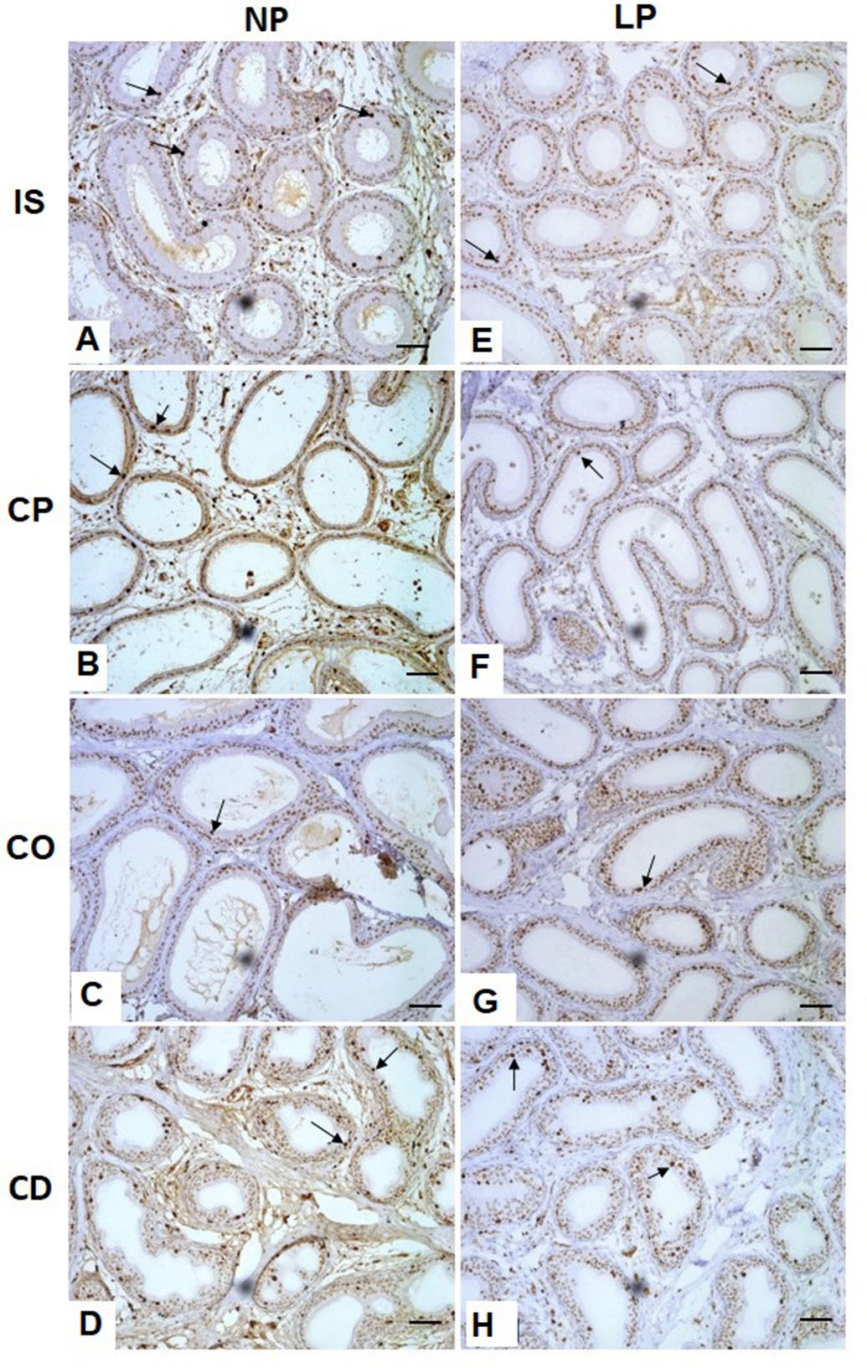
**Expression and immunolocalization of KI-67 in the rat epididymis of 44-day-old NP (*n*=5) and LP (*n*=5) groups.** Arrows indicate the positive immunoreaction for KI-67. Scale bar: 50 µm. IS, initial segment; CP, caput epididymis; CO, corpus epididymis; CD, cauda epididymis.

The immunohistochemical marker KI-67 was used to evaluate cell proliferation. Thus, it was possible to observe the KI-67 staining in some nuclei of the epithelial cells throughout the epididymis in both NP and LP groups (Fig. 3). An increase in the percentage of positive KI-67 cells was observed in IS and in the corpus of epididymis of LP animals. The cauda epididymis of LP animals showed a decrease in the percentage of positive KI-67 cells (Fig. 5).

### TP63 immunolocalization in basal cells of the epididymal duct

**Fig. 4.**
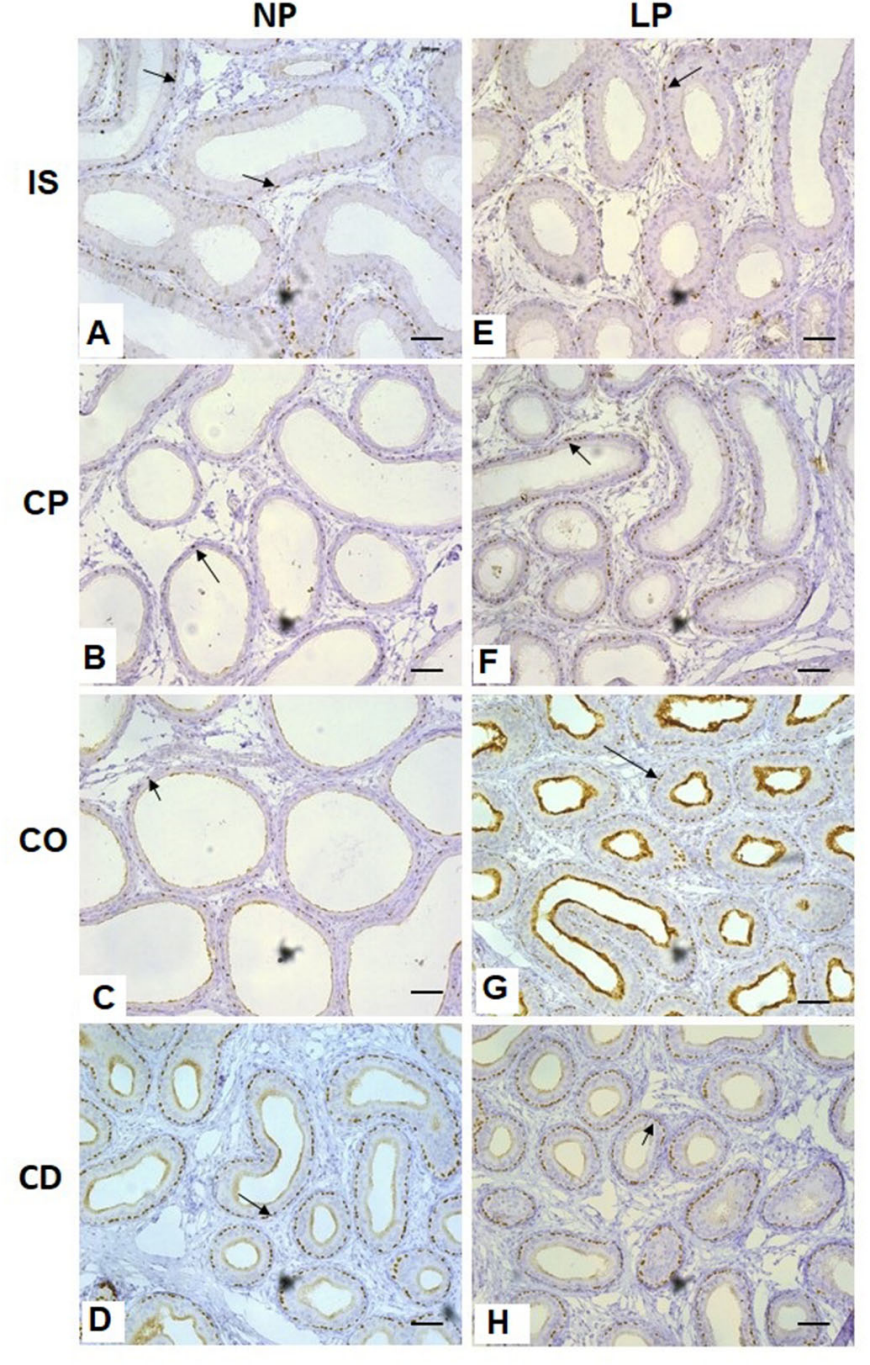
**Expression and immunolocalization of TP63 in the rat epididymis of 44-day-old NP (*n*=5) and LP (*n*=5) groups.** Arrows indicate the positive immunoreaction for TP63. Scale bar: 50 µm. IS, initial segment; CP, caput epididymis; CO, corpus epididymis; CD, cauda epididymis.

**Fig. 5.**
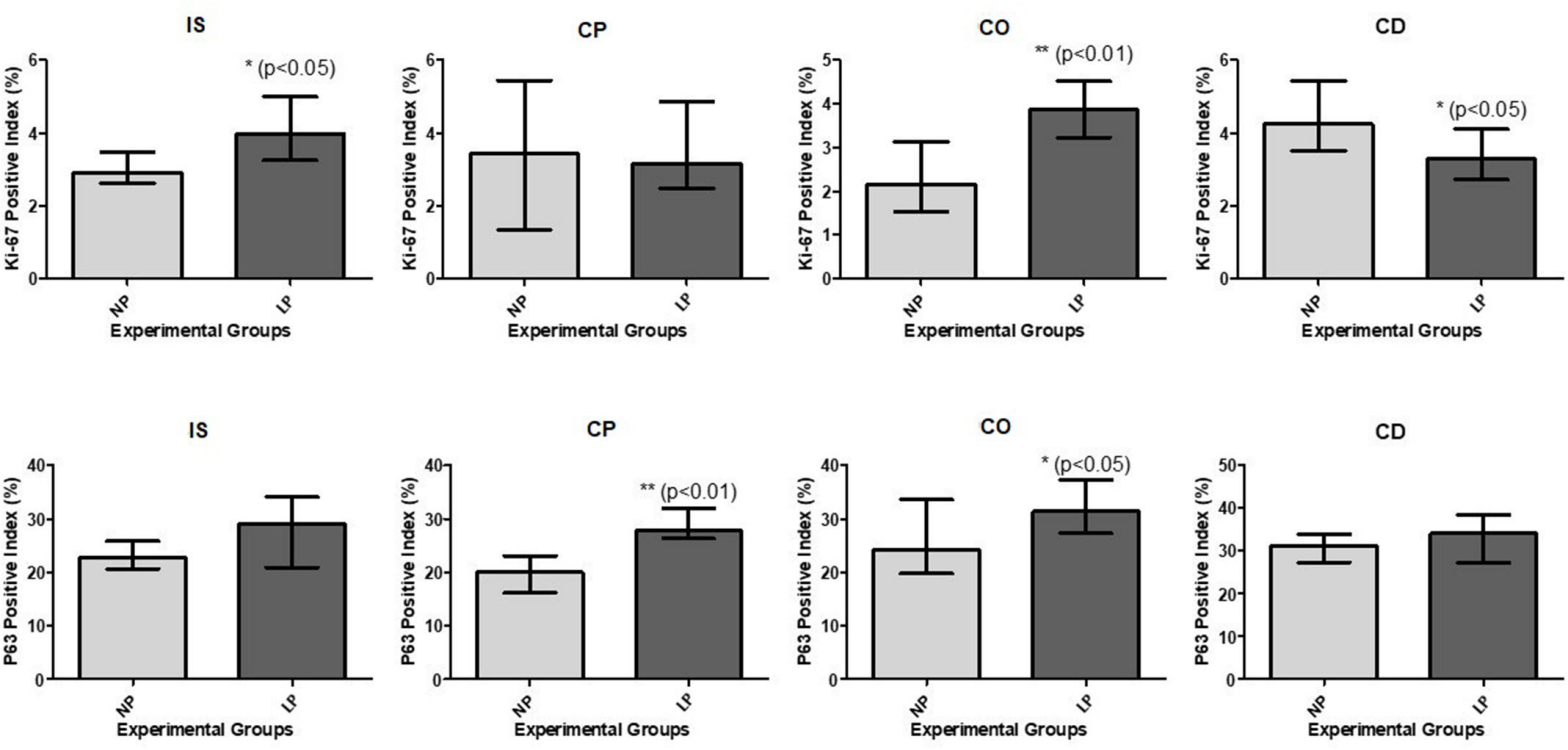
**Statistical analysis of markers KI-67 and TP 63 in the epididymal regions.** Initial segment (IS), caput (CP), corpus (CO) and cauda (CD) epididymis in the 44-day-old NP (*n*=5) and LP (*n*=5) rats. The graphs are expressed in percentage of marked cells regarding to the total number of cells of the epididymal epithelium. The data are presented as the mean±s.e.m. *P<0.05, Mann–Whitney test.

Regarding basal cells, immunolocalization was performed using anti-TP63 antibody (Fig. 4). Positive TP63 cells were observed throughout all regions of the epididymal duct in both experimental groups. An increase in the percentage of positive TP63 cells was observed in the caput and corpus epididymis of LP rats compared with these epididymal regions in NP rats (Fig. 5).

### ATPase labeling in epididymal clear cells

**Fig. 6.**
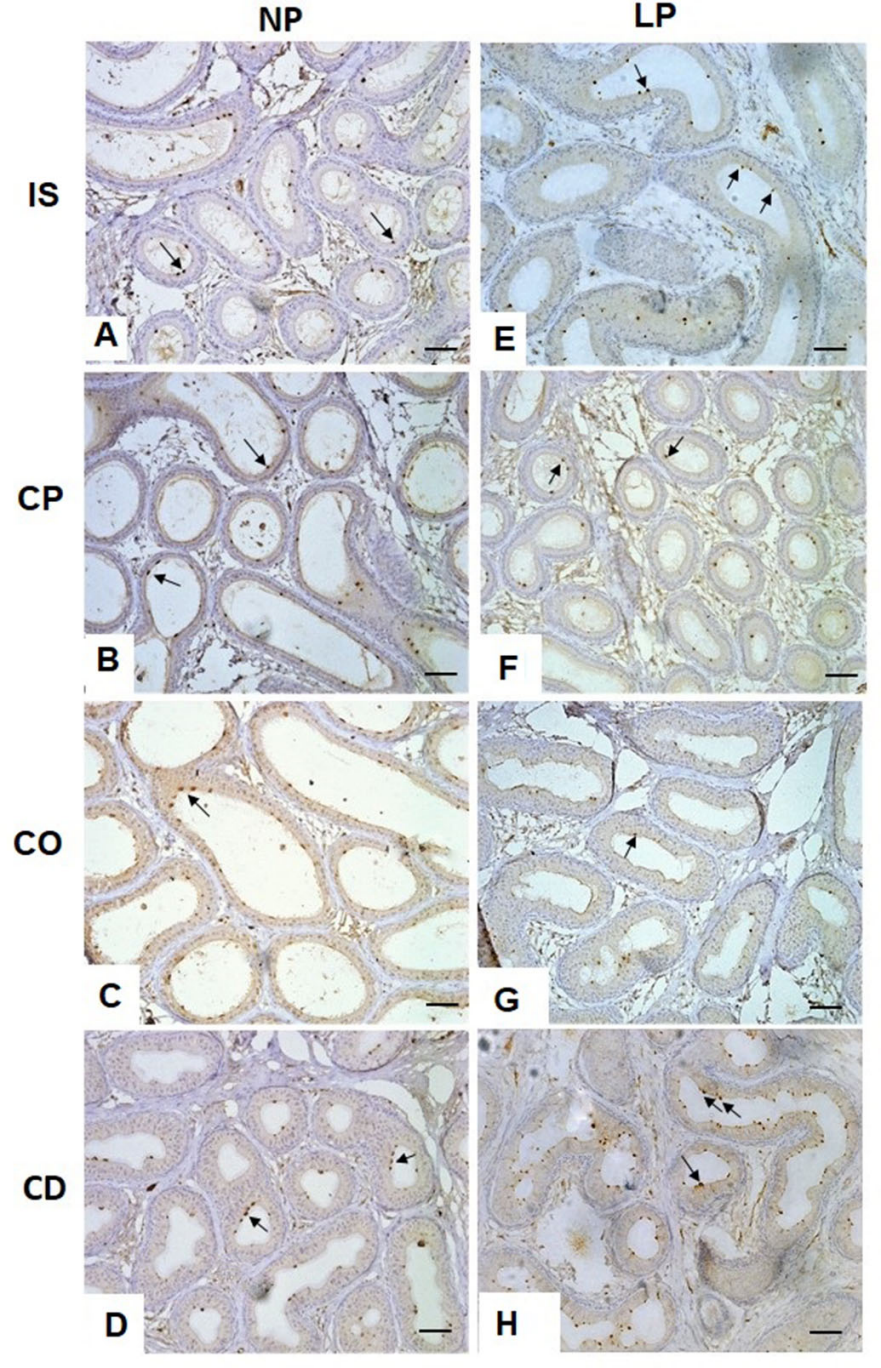
**Expression and immunolocalization of ATPase in the rat epididymis of 44-day-old NP (*n*=5) and LP (*n*=5) groups.** Arrows indicate the positive immunoreaction for ATPase. IS, initial segment; CP, caput epididymis; CO, corpus epididymis; and CD, cauda epididymis.

**Fig. 7.**
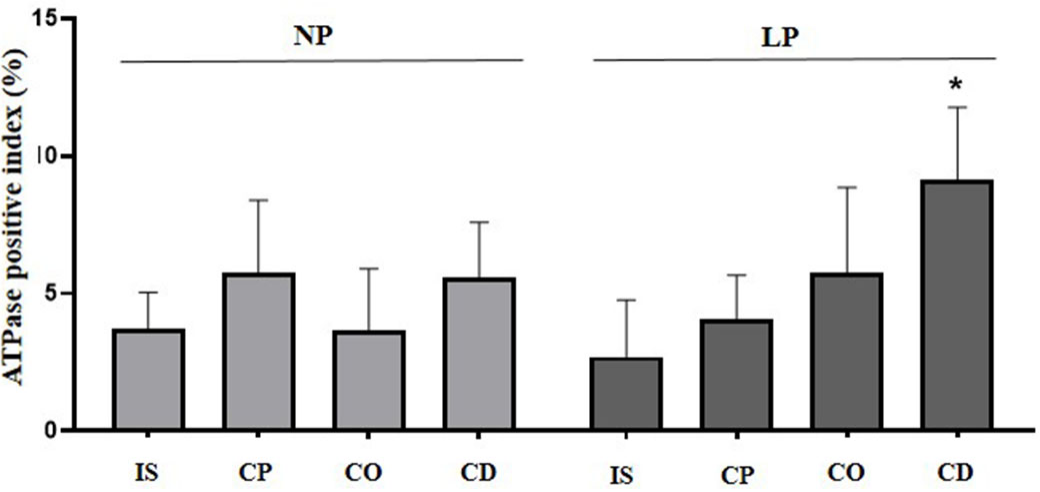
**Statistical analysis of markers ATPase in the epididymal regions.** Initial segment (IS), caput (CP), corpus (CO) and cauda (CD) epididymis in the 44-day-old NP (*n*=5) and LP (*n*=5) rats. The graphs are expressed in percentage of marked cells regarding to the total number of cells of the epididymal epithelium. The data are presented as the mean±s.e.m. *P<0.05, Mann–Whitney test.

Finally, ATPase labeling was observed in clear cells from all epididymal regions. However, an increase in positive ATPase cells was only observed in the cauda epididymis of LP animals compared with NP animals (Figs 6 and 7).

## Discussion

The present study investigated the immunolocalization of AQP9, KI-67, TP63 and ATPase in the epididymal epithelium of 44-day-old rats whose mothers were fed a low-protein diet during pregnancy and lactation. This age was chosen because 44 days of age is the final phase of epididymal differentiation and the beginning of epididymal expansion (Picut et al., 2018). At 7 and 14 days of age, maternal protein restriction leads to a delay in the differentiation of mesenchymal epididymal cells (de Mello Santos et al., 2019). This premature delay could have affected the differentiation of the epididymal epithelium in 44-day-old LP rats. At this stage, a delay in cell differentiation was observed in the epididymis of LP animals, by the decrease in AQP9 reactivity in the IS and cauda of the epididymis, and by the increase in cell proliferation, especially in the IS.

### Reduced AQP9 immunoreactivity observed in principal cells of LP group animals

According to Robaire and Hinton (2015), there are several types of epithelial cells that line the epididymis, such as principal cells, basal cells and clear cells, that are present along the different epididymal regions. Aquaporin 9 (AQP9) can be used as specific marker to highlight principal cells in the epididymis (Castro et al., 2017). The findings showed a reduction of AQP9 positive reactivity in the IS and cauda of epididymis of animals from the LP group when compared to the NP group. Since AQP9 is considered a marker of principal cell differentiation (Jun et al., 2014), it is possible to conclude that epididymal principal cells are not completely differentiated in LP animals.

AQP9 is the predominant aquaporin expressed in the mammals epididymis and plays an important role in the dynamic of reabsorption, secretion, and transportation of solutes throughout the epididymis (Badran and Hermo, 2002; Pastor-Soler et al., 2005; Da Silva et al., 2006; Domeniconi et al., 2007). A previous study by our research group, using the Western blotting technique and the same experimental protocol, reported a decrease in AQP9 protein levels in the proximal regions of the epididymis of LP animals at 44 days of age (Cavariani et al., 2019). Arrighi et al. (2010) also observed a reduction in AQP9 expression in the epididymis of adult rats subjected to malnutrition during the perinatal period. AQP9 expression is modulated by testosterone, and the proximal regions of epididymis are more sensitive to testosterone variations. These features could also explain the lower AQP9 expression in the epididymal proximal regions of LP rats since it is related to the decrease in serum testosterone in LP animals at this age (Cavariani et al., 2019).

### Rise in TP63-positive cell percentage in caput and corpus epididymis of LP rats

Basal cells are another epididymal cellular type and are present in the epididymal epithelium lining of mammals (Arrighi, 2014). In this study, the TP63 marker was used to immunoidentify basal cells. Basal cells were observed along the epididymis in both studied groups (NP and LP), similar to that reported by Arrighi (2014), who claimed that these epididymal cellular types appear in all epididymal regions and can collaborate in the building up of the blood epididymis barrier through cell adhesion molecules. Therefore, in this study, TP63 was more expressed in LP rats. Although TP63 increased significantly only in the caput and corpus regions, there was a slight increase in TP63 staining in all regions of the epididymis in the LP group. These results indicate that these cellular types play a pivotal role in the restructuring process of the epididymal epithelium in offspring from mothers subjected to protein restriction during pregnancy and lactation, since the basal cells are involved in the cellular renovation process (Sharma et al., 1986). The role of basal cells has been widely discussed, and some authors have shown that this cellular type could be involved in cell-to-cell interactions as well as working as luminal sensors to regulate the activity of principal and clear cells. Another hypothesis for the role of basal cells is that they likely contribute to the blood epididymis barrier through molecules of cellular adherence, and as a consequence, they would be involved in the immunological control of spermatozoa, which are outsiders in the environment where they are produced (Arrighi, 2014).

### Increase in ATPase-positive cells noted in the cauda epididymis of LP animals

Clear and principal cells are involved in maintaining an acidic luminal pH throughout the epididymis. The luminal acidification is dependent on processes such as the reabsorption of bicarbonate and the secretion of protons. Reabsorption of bicarbonate is performed by the principal cells in the epididymal IS, and the secretion of protons is one of the functions assigned to clear cells, which are more abundant in the epididymal distal regions, especially in the cauda epididymis, and in the proximal part of the vas deferens (Shum et al., 2011; Breton et al., 2016). This study showed an increase in the number of ATPase-positive cells in the cauda epididymis of rats subjected to maternal protein restriction. The increase in V-ATPase expression in the apical region of the epididymal epithelium is related to the increase in the secretion of protons, which are chained in response to luminal pH variations (Brown and Breton, 2000; Pastor-Soler et al., 2003). The response of the epididymal epithelium to lower the luminal pH in the cauda is because this region is essential in the maintenance of quiescent spermatozoa during their maturation and storage in the epididymis (Pastor-Soler et al., 2005; Shum et al., 2011).

### Differential KI-67 staining reveals cell proliferation variations across epididymal regions in NP and LP animals

KI-67 is a nuclear antigen related to cellular proliferation that participates in cellular mitosis and is used as a marker of cells that have not entered the G0 (MacCallum and Hall, 2000; Scholzen and Gerdes, 2000; Takagi et al., 2016). KI-67 can be used as a marker of cellular proliferation in its normal or tumoral state (Li et al., 2015; Coşarcã et al., 2016). The findings showed a significant increase of KI-67-positive cells in IS and corpus epididymis in LP animals, which means that there was an increase in cellular proliferation during the differentiation period when is expected to decrease the KI-67 proliferative index. At this stage, the Ki-67 proliferative index is expected to decrease because the cells exit the cell cycle and enter a differentiated postmitotic G0 stage (Jun et al., 2014). In contrast, in the cauda of the epididymis of LP rats, cell proliferation decreased, indicating that the delay in differentiation is more characterized in the proximal regions. In addition, the increase in normal cellular proliferation observed in LP rats could be related to the attempt of the epididymal epithelium to be restructured and reestablished.

Based on the findings of this study, 44-day-old rats of the LP group presented an epididymal epithelium with a different phenotype from that of the NP group. Additionally, the delay in the differentiation of mesenchymal cells observed at the beginning of the post-natal period persisted around 44 days of post-natal life. At 44 days of age, the epithelium is expected to be completely differentiated, but LP animals show a delay in epithelial differentiation. It is worth highlighting that the observed alterations seem like an attempt by the epithelium lining to reestablish itself before the reaching of sexual maturity by the animal.

## MATERIALS AND METHODS

### Animals and experimental design

Adult female (60 days of age, *n*=38) and male (90 days of age, *n*=20) Wistar rats were obtained from the Central Biotherium, São Paulo State University (UNESP), campus of Botucatu, São Paulo, Brazil. The animals were housed in polyethylene cages (43×30×15 cm) lined with an autoclaved pine sawdust substrate under controlled conditions of temperature (22±2°C) and light (12 h light/dark cycle). Balanced rat chow and filtered tap water were provide *ad libitum*. The experimental procedures were approved by the Ethical Committee on Animal Use (number 797, CEUA), Institute of Biosciences of Botucatu, UNESP.

For mating, two sexually receptive females and one breeder male rat at 95 days of age were kept in maternity boxes overnight. In the following day, vaginal smears were performed. The presence of sperm in the vaginal smear indicated pregnancy, and this day was considered gestational day 0 (GD 0). Pregnant females were randomly allocated into two experimental groups: The normal-protein (NP) group (*n*=10) and the low-protein (LP) group (*n*=10). The NP females were fed a normal-protein diet (17% protein), during gestation and lactation, while the LP females were fed a low-protein diet (6% protein), during gestation and lactation. Both diets were provided from Pragsoluções Biociências (Jaú, São Paulo, Brazil). Both groups received their respective diet *ad libitum* (Table 1).

**Table 1.** Composition of the diets offered to the rats during the gestation and lactation

Components*	NP (17% protein)	LP (6% protein)
Casein (84% of protein)**	**202.00**	**71.50**
**Cornstarch**	**397.00**	**480.00**
**Dextrin**	**130.50**	**159.00**
**Sucrose**	**100.00**	**121.00**
**Soy oil**	**70.00**	**70.00**
**Fiber (microcellulose)**	**50.00**	**50.00**
Mineral Blend***	**35.00**	**35.00**
Vitamin Blend***	**10.00**	**10.00**
**L-cystine**	**3.00**	**1.00**
**Choline chloride**	**2.50**	**2.50**

*Diet for rodent during gestation – AIN-93G. **Corrected values according to protein content in casein. ***Following AIN-93G.

After birth, eight pups per litter (males) were maintained with each mother to ensure equal availability of nourishment. The NP and LP diets were managed until the offspring were weaned at PND 21. After weaning, the NP and LP male offspring received the standard diet for rodents until the age of 44 (NP, *n*=10, LP, *n*=10) days (Fig. 1). The male offspring were euthanized at 44 days of age. This age was chosen since at 44 days of age represents the final phase of epididymal differentiation and the beginning of epididymal expansion (Picut et al., 2018). The animals were weighed, and the epididymis were collected and also weighed (these data have already been discussed by Cavariani et al., 2022).

### Immunohistochemistry

Epididymal samples from the NP (*n*=10) and LP (*n*=10) rats were subjected to antigen retrieval in a humid environment (electric pot) at 100°C in Tris/0.1 M EDTA pH 9.0 for 30 min. After being washed in distilled water, the sections were subjected to the blocking of endogenous peroxidase (3% hydrogen peroxide in methanol) for 15 min. To block nonspecific binding, the slides were incubated with 3% skimmed milk in PBS for 1 h. Then, the sections were incubated overnight at 4°C with primary antibodies, at dilution 1:200 in BSA 1% as described on Table 2. After incubation with the primary antibodies, the materials were washed in PBS and then incubated with HRP secondary antibody at dilution 1:100 in BSA 1% for 1 h at room temperature and subsequently visualized with DAB chromogen (3,3′-diaminobenzidine tetrahydrochloride, Sigma-Aldrich, St. Louis, MO, USA) and counterstained with hematoxylin for 1 min. We observed a marking pattern in proteins in different cell types, specific from each epididymis region. Four epididymal regions were considered to analyze the results: initial segment (IS), caput (CP), corpus (CO) and cauda (CD) (Turner et al., 2003; Domeniconi et al., 2016). The negative control by suppression of the primary antibody was used to confirm the specific reaction.

**Table 2.** Antibodies immunohistochemistry

**Primary antibodies**	**Cell marker**	**Code**	**Source**
AQP9	Principal cells	AQP91-A	α Diagnostic
KI-67	Cellular proliferation	ab15580	Abcam
TP63	Basal cells	sc-8343	Santa Cruz
V-ATPase	Clear cells	sc-271832	Santa Cruz

After performing immunohistochemistry, the slides were pre-identified for analysis of the ‘blind’. The description of the results obtained by the AQP9 reaction were made by comparing the intensity of the reaction between the two experimental groups, and were carried out by Colonheze and confirmed by Dr Domeniconi. The cells that expressed KI-67, TP63 and ATPase were counted using the Pannoramic Viewer program. The number of marked cells was divided by the total number of epididymal cells. Ten transverse sections of epididymal tubules in each of the epididymal regions were performed from each of the five animals of each group (NP and LP). After, the average and the value found multiplied by 100 for each animal were calculated according to Peixoto et al. (2016). The data were represented by the median value followed by the interquartile range. For the statistical analysis, we used the non-parametric test by Mann–Whitney.

### Statistical analysis

For the statistical analyses, GraphPad Prism (version 5.00, Graph Pad, Inc., San Diego, CA, USA) was used. The comparisons between groups NP and LP were made using Mann–Whitney test for nonparametric data. The data are presented considering the statistical significancy of *P*<0.05.
